# High rates of sexual violence by both intimate and non-intimate partners experienced by adolescent girls and young women in Kenya and Zambia: Findings around violence and other negative health outcomes

**DOI:** 10.1371/journal.pone.0203929

**Published:** 2018-09-13

**Authors:** Sanyukta Mathur, Jerry Okal, Maurice Musheke, Nanlesta Pilgrim, Sangram Kishor Patel, Ruchira Bhattacharya, Nrupa Jani, James Matheka, Lunda Banda, Drosin Mulenga, Julie Pulerwitz

**Affiliations:** 1 Population Council, Washington, DC, United States of America; 2 Population Council, Nairobi, Kenya; 3 Population Council, Lusaka, Zambia; 4 Population Council, New Delhi, India; UNAIDS, UNITED STATES

## Abstract

**Background:**

While links between intimate-partner violence (IPV) and HIV risk have been established, less is known about violence perpetrated by people other than intimate partners. In addition, much of the research on IPV has been conducted with adults, while relatively little is known about violence experienced by adolescent girls and young women (AGYW). We examined experiences of sexual violence and associated sexual and mental health among AGYW in Kenya and Zambia.

**Methods:**

Using cross-sectional surveys with women aged 15–24 years, we assessed experience of partner sexual violence among respondents who reported a boyfriend/husband in the last 12 months (Kenya N = 597; Zambia N = 426) and non-partner sexual violence among all respondents (Kenya N = 1778; Zambia N = 1915). We conducted logistic regression analyses to examine experiences of sexual violence and health outcomes.

**Results:**

Sexual violence from intimate partners over the last year was reported by 19.1 percent of AGYW respondents in Kenya and 22.2 percent in Zambia; sexual violence from non-partners was reported by 21.4 percent in Kenya and 16.9 percent in Zambia. Experience of sexual violence was associated with negative health outcomes. Violence from non-partners was associated with increased odds of STI symptoms and increased levels of anxiety and depression. Results were similar for violence from partners, although only significant in Kenya. While sexual violence from a non-partner was associated with increased HIV risk perception, it was not associated when the violence was experienced from an intimate partner.

**Conclusions:**

AGYW reported high levels of sexual violence from both intimate partners and non-partners. These experiences were associated with negative health outcomes, though there were some differences by country context. Strengthening sexual violence prevention programs, increasing sexual violence screening, and expanding the provision of post-violence care are needed to reduce intimate and non-partner violence and the effects of violence on AGYW.

## Introduction

Violence against women is a global epidemic with implications for women’s health and development outcomes. The World Health Organization (WHO) estimates that approximately one third of women globally have experienced some kind of violence (physical and/or sexual) from a partner or non-partner in their lifetime [1]. There are increasingly robust regional or age-specific estimates about violence from intimate partners. For instance, the lifetime prevalence of intimate partner violence is 29 percent among 15–19-year-olds and 32 percent among 20–24-year-olds [1]. However, less in known about violence from non-partners (men who are not boyfriends/husbands/regular partners) [1]. Data on non-partner violence is often hampered by little research on the topic, reluctance to report due to stigma surrounding sexual violence, and lack of standardization in measurement [2]. For instance, sexual violence estimates usually capture domestic or intimate-partner violence from ever-married or cohabitating women, whereby limiting our understanding of sexual violence that occurs outside of partnerships [3]. Thus, while the available data show that 7.2 percent of women aged 15 years or older experience non-partner sexual violence globally [1], limited age-specific data means that adolescent girls and young women’s (AGYW’s) experiences of non-partner sexual violence remain largely undocumented [1–2]. In this paper, we examine experiences of sexual violence from intimate partners (IP) and non-partners among AGYW in two country contexts in sub-Saharan Africa.

Experience of violence, sexual violence in particular, from IPs and non-partners can have prolonged health impacts on women’s lives [1, 4–7]. A systematic review of 28 studies demonstrated a significant association between intimate partner violence (IPV) and HIV [8]. Women who experience physical and/or partner sexual violence are 1.5 times more likely to acquire sexually transmitted infections (STIs), and in sub-Saharan Africa women are 1.5 times more likely to acquire HIV [ibid]. A prospective study in South Africa, for instance, showed that IPV was significantly associated with higher levels of subsequent HIV risk [5]. Similarly, available data show that non-partner violence has negative implications for physical and mental health outcomes in the short and long term, contributing to the burden of poor health of affected women [9–10]. Survivors of non-partner violence are 2.3 times more likely to have alcohol use disorders and 2.6 times more likely to experience depression or anxiety [1]. It has also been shown that sexual violence by strangers is more violent and has a higher risk of injury and infection with HIV and other STIs [2, 9–10]. Despite the mounting evidence on the association between sexual violence and negative health outcomes, research on non-partner sexual violence and associated health risks, among AGYW in particular, is limited in the sub-Saharan Africa region [3, 11] and is the focus of this analysis.

We present evidence from Kenya and Zambia. Both the Kenyan and Zambian governments recognize violence against women as a significant issue and have enacted domestic policies and ratified international treaties to stem violence against women and children. Further, a substantial proportion of AGYW are at elevated risk for HIV and STIs in the two countries [12]. In Kenya and Zambia, 1.1 percent and 4.8 percent of adolescent girls, and 4.6 percent and 11.2 percent of young women, respectively, are living with HIV [13–14]. Given that sexual violence has been shown to be intimately linked with HIV, an important first step in mitigating against HIV is an improved understanding of the occurrence of IP and non-partner sexual violence and its associated health effects, especially among AGYW [2].

In this paper, we focus on adolescent girls and young women’s experiences of IP and non-partner sexual violence. In doing so, we estimate the prevalence of IP and non-partner sexual violence, as well as its associations with HIV risk perception, STI, and mental health among Kenyan and Zambian AGYW. Understanding the magnitude of IP and non-partner sexual violence among AGYW is critical to developing contextually-relevant policies and interventions to prevent violence against young women and provide appropriate interventions to mitigate against the adverse health effects.

## Methods

### Study setting

This study draws on cross-sectional surveys with AGYW, aged 15–24 years, in Kenya and Zambia. The data for this analysis was collected as part of a larger implementation science research project that focuses on understanding HIV risk among AGYW and exploring the effectiveness of the US President’s Emergency Plan for AIDS Relief (PEPFAR)-supported DREAMS Partnership, a program focused on reducing HIV risk and incidence amongst AGYW and their male partners [15]. Sites for DREAMS programming were selected by PEPFAR in consultation with local government representatives and other stakeholders to reflect areas of high HIV prevalence in each country. In Kenya, the study was conducted in an informal settlement and a peri-urban area in Kisumu County. In Zambia, the study was conducted within the urban wards of Lusaka and Ndola. In general, the study sites are also characterized by high poverty levels and a densely populated and/or rapidly growing population. This analysis uses cross-sectional data collected from AGYW towards the beginning of the DREAMS program implementation timeline.

### Sample size and sampling strategy

Eligible participants for the surveys were females aged 15–24 years who were residing in the program catchment area, had an intention of staying in the area for the subsequent year, and who agreed to participate in the survey. We randomly sampled AGYW from both program participant lists and the surrounding communities (i.e., multi-stage household random samples) to ensure representativeness of responses. In Kenya, a total of 1,778 AGYW were interviewed from October 2016 to February 2017. In Zambia, a total of 1,915 AGYW were interviewed from November 2016 and April 2017. Refusal rates were minimal, with 20 potential respondents in Kenya and 33 in Zambia refusing to participate due to lack of parental consent or limited time availability. If the eligible respondent was not available, three attempts were made to contact and schedule an interview.

### Study procedures

Before administering the quantitative survey, interviewers obtained informed consent from study participants (or parental consent and respondent assent, as appropriate). Following consenting procedures, the interviews were conducted by trained female interviewers using a tablet for data collection. The interviews were conducted in private convenient locations, usually at a community space used by the DREAMS program or in a private space within or around the respondent’s home. All questions were administered by the interviewer in a local language of the respondents choosing (English, Kiswahili, Luo, and English in Kenya, and English, Bemba, or Nyanja in Zambia). Participants were offered referrals (if requested) for medical services in nearby locations at Ministry of Health clinics. Participants in Kenya were reimbursed KSH300 (approximately US$3), and ZMW50 Kwacha (approximately US$5) in Zambia, based on the amount recommended by the relevant ethical review boards.

### Key measures and data analysis

A comprehensive knowledge, attitudes, and behaviors survey was administered, including specific questions on the key *explanatory variables* for this analysis—experience of sexual violence from IP and non-partners. In both countries, representatives of local program partners were consulted during the process of designing the survey. Questions on sexual violence from IPs and non-partners were taken from the WHO’s Violence Against Women scale, which has been adapted and used in southern Africa [5, 16]. Questions on IPV were asked of all AGYW who reported having a boyfriend/husband/partner as their main sexual partner in last the 12 months (Cronbach’s alpha: 0.85 in Kenya and 0.74 in Zambia). We constructed a dichotomous variable of experience of sexual IPV from a set of three of questions on frequency of sexual IPV: “physically forced into sex”, “threatened into sex”, and “forced to do any other sexual act”. If AGYW experienced any act of sexual violence in last the 12 months, it was coded as “experienced.” If experience of violence was nil in all the three questions, then the variable was coded as “not experienced.” Questions on non-partner sexual violence were asked of all AGYW, and included four questions on sexual violence from men who were not boyfriends/husbands/partners (Cronbach’s alpha: 0.53 in Kenya and 0.54 in Zambia). These included: “forced or persuaded you to have sex against will”, “tried to force you to have sex”, “forced to have sex against will you were too drunk or drugged to refuse”, and “two or more men force you to have sex with them at the same time.” If AGYW experienced any of these acts of sexual violence in the last 12 months, it was coded as “experienced,” if experience of violence was nil in all the four questions, it was coded as “not experienced”.

Three *outcome variables* were used in this analysis. First, we examined self-perception of HIV risk as a proxy indicator of high risk for HIV acquisition, as confirmed in previous studies with young people in the region [17]. AGYW were asked how likely it was that they were exposed to HIV and measured on a 4-point scale from “very likely” to “not at all.” We created a dichotomous measure where respondents who noted “very likely” and “somewhat likely” or “don’t know” were coded “high risk” and the rest were coded as “low risk”, excluding respondents who were currently living with HIV. Second, we examined the experience of STI symptoms, adapting standard Demographic Health Survey (DHS) measures [18]. We constructed a dichotomous measure using self-report of STI symptom experience in the last 6 months, asking about genital ulcers, vaginal discharge, painful urination, and genital warts. Respondents were coded as “1” if they had reported an STI or “0” if they did not report any STI symptoms in the last 6 months. Finally, we used the PHQ-4 mental health scale to measure anxiety and depression [19]. AGYW were asked a set of four questions on how often they had feelings such as “nervousness or anxiety”, “not being able to stop or control worrying”, “down, depressed, or hopeless”, and “lack of interest or pleasure in doing things” in the past two weeks. Response options were 0, “never”; 1, “several days”; 2, “more than half the days”; and 3, “all the days.” Responses in all four questions were added to create a composite score ranging from 0 to 12. Following the convention for the scale, a dichotomous variable was created recoding 0–5 scores as 0 or “Low anxiety and depression” and 6–12 score as 1 or “High anxiety and depression” (Cronbach’s alpha: 0.80 in Kenya and 0.78 in Zambia).

Descriptive analysis was conducted to examine key respondent characteristics for AGYW in Kenya and Zambia. Initial analysis revealed that experiences of sexual violence were similar among DREAMS program participants and non-program participants (for instance in Zambia, 20 percent of DREAMS program participants and 23 percent of AGYW from the surrounding community reported experience of sexual violence in the last year); thus, in this analysis we examine the experience of sexual violence and associated health outcomes among all AGYW. Subsequent bi-variate analysis was carried out to explore the experiences of IP and non-partner sexual violence by selected indicators related to AGYW socio-demographic characteristics, sexual behavior, and the outcome variables. Multivariable logistic regression analyses were conducted to examine the association of IP and non-partner sexual violence with self-perception of HIV risk among AGYW, STI experience, and mental health. The results are presented as unadjusted odds ratios and adjusted odds ratios (AORs), controlling for socio-demographic factors known to be associated with experience of violence and HIV risk: age, educational attainment, current schooling status, marital status, household socio-economic status (based on household construction/infrastructure), and orphanhood status.

### Ethical approval

The study protocols were reviewed and approved by the Population Council Institutional Review Board (IRB), as well by the Kenyatta National Hospital/University of Nairobi Ethics and Research Committee and National Commission for Science Technology and Innovation in Kenya, and ERES CONVERGE IRB and the National Health Research Authority in Zambia. Written informed consent from study participants (or parental consent and respondent assent, as appropriate) was received from all respondents before proceeding with the survey.

## Results

### Characteristics of study participants and experiences of sexual violence

Table 1 presents characteristics of the study participants in Kenya and Zambia. The mean age of respondents was similar in Kenya and Zambia, at 19.3 and 19.4 years, respectively. More AGYW in Kenya attained primary education level than Zambia (48.8 percent vs.19.9 percent), but more AGYW in Zambia attained secondary (66.1 percent vs. 46.4 percent) and tertiary (14.0 percent vs. 4.8 percent) education than in Kenya. In both countries, more than half of the AGYW were in school. Similarly, more than two-thirds of the AGYW in the two countries were not married, whereas almost twice as many respondents in Kenya than Zambia were sexually active (46.8 percent vs. 26.8 percent). In terms of their household background, slightly more than one-third of the girls in both Kenya and Zambia were single orphans, and more than half in Zambia, and almost half in Kenya, were not orphans. In terms of socio-economic status, most girls in both countries were either from low socio-economic households (33.8 percent in Kenya and 36.5 percent in Zambia) or from medium socio-economic households (36.5 percent in Kenya and 43.4 percent in Zambia).

**Table 1:** Socio-demographic characteristics of respondents, Kenya and Zambia.

Characteristics	Kenya (N = 1778)	Zambia (N = 1915)
**Age**	%	%
Mean age (in years)	19.3	19.4
15–19 years	50.3	50.0
20–24 years	49.7	50.0
**Educational attainment**		
Primary	48.8	19.9
Secondary	46.4	66.1
Tertiary	4.8	14.0
**Currently in school**		
Yes	57.0	55.8
No	43.0	44.2
**Marital status**		
Never married	65.9	87.1
Married/cohabiting	30.2	9.2
Formerly married	3.9	3.7
**Sexually active**		
Yes	46.8	26.8
No	53.2	73.2
**Household socio-economic status**		
Low	33.80	36.5
Medium	36.5	43.4
High	29.7	20.1
**Orphanhood status**		
Not an orphan	49.0	57.3
Single orphan (lost 1 parent)	34.3	32.2
Double orphan (lost both parents)	16.7	10.5

Table 2 presents experience of sexual violence and sexual and mental health outcomes among respondents. In the last 12 months, 19 percent of AGYW in Kenya and 23 percent in Zambia reported sexual violence from an IP. The reported occurrence of sexual violence from non-partners in the last 12 months was also high, at 21 percent in Kenya and 17 percent in Zambia. Overall, access to post-violence services such as counselling, treatment, and support was low in both Kenya and Zambia. The majority of the AGYW in Kenya (85.3 percent) and Zambia (64.9 percent) reported being HIV negative. One third of the respondents in Zambia did not know their HIV status, while 16 percent in Kenya and 9 percent in Zambia perceived they were at risk of HIV. A high proportion of AGYW reported experiencing STI symptoms in the last six months in both countries (approximately 18 percent in Kenya and 15 percent in Zambia). Recent reports of feeling anxious or depressed were low among the respondents in both Kenya and Zambia (4 percent and 9 percent, respectively).

**Table 2:** Experience of violence and sexual and mental health outcomes, Kenya and Zambia.

Characteristics	Kenya	Zambia
	%	%
**Experienced sexual violence from intimate partner in the last 12 months (among those with partners)**	(n = 597)	(n = 426)
Yes	19.1	22.8
No	80.9	77.2
	(N = 1778)	(N = 1915)
**Experienced sexual violence from non-intimate partner in the last 12 months**		
Yes	21.4	16.9
No	78.6	83.1
**Use of violence care services, counseling, and/or prevention in the last year**		
Yes	12.2	7.0
No	87.8	92.9
**HIV status**		
HIV+	4.0	1.9
HIV-	85.3	64.9
Don’t know	10.7	33.2
**Perceived likelihood of being exposed to HIV**		
Very/Somewhat Likely/Don’t know	16.1	9.0
Not at all/Unlikely	83.9	91.0
**STI symptoms in the last 6 months**		
Yes	17.7	14.6
No	82.3	85.4
**Anxiety & depression in the last 2 weeks**		
Low anxiety & depression (0–5)	96.3	90.6
High anxiety & depression (6–12)	3.7	9.4

In exploratory bivariate analyses (results not presented), we found that, as has been shown in previous research, lower educational attainment, marital experience, and household socio-economic status were significantly associated with experience of IP sexual violence in Kenya, but not Zambia. For non-partner sexual violence, educational attainment and marital status were significantly associated with sexual violence in both countries.

### Sexual violence and HIV risk, STI experience, and mental health

Tables 3 and 4 present bivariate associations between experience of sexual violence from IPs and non-partners in the last 12 months and health outcomes. In Kenya, IPV was associated with not knowing their HIV status, STI symptoms, and high levels of anxiety and depression. In Zambia, only HIV status was significantly associated with experience of IPV; 57.1 percent of HIV-positive respondents had experienced sexual violence from IP, compared to those who did not know their HIV status (23.6 percent) or were HIV-negative (21.3 percent). In both countries, non-partner sexual violence was significantly associated with STI experience, increased risk perception, and higher anxiety and depression.

**Table 3:** Bivariate associations between experience of IP sexual violence and health outcomes, Kenya and Zambia.

	KENYA			ZAMBIA		
Characteristics	N	%	p-value	N	%	p-value
	597	19.1		426	22.2	
**STI symptoms**			**<0.001**			0.119
Yes	145	31.7		86	29.1	
No	452	15.0		340	21.2	
**HIV status**			**0.018**			**0.007**
HIV+	34	14.7		14	57.1	
HIV-	545	18.5		338	21.3	
Don’t know	18	44.4		72	23.6	
**Self-perception of HIV-risk**			0.029			0.328
Somewhat likely	95	27.4		44	15.9	
Unlikely	464	17.7		367	22.3	
**Anxiety & depression**			**0.001**			0.252
Low anxiety & depression	569	17.9		386	22.0	
High anxiety & depression	28	42.9		40	30.0	

**Table 4:** Bivariate associations between experience of non-partner sexual violence and health outcomes, Kenya and Zambia.

	KENYA			ZAMBIA		
Characteristics	N	%	p-value	N	%	p-value
	1778	21.4		1915	16.9	
**STI symptoms**			**<0.001**			**<0.001**
Yes	315	31.1		279	27.2	
No	1463	19.3		1636	15.2	
**HIV status**			0.1			0.247
HIV+	71	16.9		36	13.8	
HIV-	1516	22.3		1241	17.9	
Don’t know	191	16.2		636	15.0	
**Self-perception of HIV-risk**			**<0.001**			**<0.001**
Somewhat likely	271	34.0		169	32.0	
Unlikely	1410	19.7		1709	15.5	
**Anxiety & depression**			**0.002**			**<0.001**
Low anxiety & depression	1713	20.8		1732	15.9	
High anxiety & depression	65	36.9		183	26.8	

Tables 5 and 6 present multi-variate analysis that explore the association between experiences of sexual violence and sexual health and mental health outcomes. Both raw and adjusted odds ratios are presented, controlling for key socio-demographic characteristics (i.e., age, educational attainment, schooling status, household socioeconomic status, and orphanhood). In Kenya, AGYW who report sexual violence from IPs had increased odds of experiencing STI symptoms (AOR: 2.67, CI: 1.70–4.18) and higher levels of anxiety and depression (AOR: 2.84, CI: 1.25–6.44). In Zambia, there was no significant association between IP sexual violence and either of the three outcome variables shown in Table 5.

**Table 5:** Experience of sexual violence with IPs and association with sexual and mental health outcomes, Kenya and Zambia.

	KENYA			ZAMBIA		
	OR (95% CI)	AOR (95% CI)	P-Value	OR (95% CI)	AOR (95% CI)	P-Value
Likelihood of exposure to HIV	1.75 (1.05–2.92)	1.78 (1.04–3.04)	0.034	0.66 (0.28–1.53)	0.69 (0.28–1.68)	0.414
Experience of STI symptoms in the last 6 months	2.62 (1.69–4.05)	2.67 (1.70–4.18)	<0.001	1.53 (0.90–2.60)	1.53 (0.88–2.66)	0.134
Level of Anxiety and depression	3.43 (1.57–7.48)	2.84 (1.25–6.44)	0.012	1.53 (0.74–3.11)	1.51 (0.73–3.14)	0.271

Multi-variate analysis (Table 6) measuring association between non-partner sexual violence and the three outcome variables showed a significant association with each of the three outcomes variables in both Kenya and Zambia. AGYW who reported sexual violence from non-partners had approximately twice the odds of perceiving themselves at HIV risk, have had an STI experience, and had higher levels of anxiety and depression.

**Table 6:** Experience of sexual violence with non-partners and association with sexual and mental health outcomes, Kenya and Zambia.

	KENYA			ZAMBIA		
	OR (95% CI)	AOR (95% CI)	P-Value	OR (95% CI)	AOR (95% CI)	P-Value
Likelihood of exposure to HIV	2.10 (1.58–2.79)	1.99 (1.49–2.67)	<0.001	2.55 (1.81–3.63)	2.00 (1.38–2.88)	<0.001
Reported STI symptoms in the last 6 months	1.88 (1.43–2.47)	2.06 (1.55–2.74)	<0.001	2.10 (1.56–2.82)	1.92 (1.42–2.61)	<0.001
Level of anxiety and depression	2.22 (1.32–3.72)	2.10 (1.23–3.57)	0.006	1.94 (1.36–2.75)	1.83 (1.84–2.64)	<0.001

## Discussion

This study adds to the evidence base on the prevalence and consequences of sexual violence among adolescent girls and young women aged 15–24 years in Kenya and Zambia. While there is increasing documentation of domestic or intimate partner violence among adult populations, our study unpacks experiences of sexual violence from intimate partners and non-partners among AGYW. And within the context of generalized HIV epidemics in Kenya and Zambia, we assessed how sexual violence is associated with HIV-related risk factors (STI experience, poor mental health, and increased risk perception).

Existing evidence suggests that sexual and gender-based violence is common in the East and Southern Africa region and affects a substantial proportion of women across the region [20]. The DHS data show that 2.7 percent and 4.2 percent of adolescent girls (15–17 years), and 7.0 percent and 10.2 percent of young women (20–24 years) have experienced sexual violence from IPs in the past 12 months in Kenya and Zambia, respectively [13–14]. The evidence we present here from Kenya and Zambia suggests that the magnitude of IP and non-partner sexual violence among AGYW may be much higher than previously recorded. Notable differences in the reporting of IP and non-partner sexual violence have been documented globally. Generally, the reports of IPV are much higher than non-partner violence, potentially this difference is due to widespread stigma associated with non-partner sexual violence resulting in underreporting of this type of violence [1]. We find high reports of non-partner sexual violence among AGYW in Kenya and Zambia. Even still, this may present a severe under-reporting of sexual violence [21] and behooves urgent examination and public health response for the sexual coercion that young women might be facing from a range of partners.

Our data confirm that for AGYW, sexual violence experiences are significantly associated with negative health outcomes. In most of sub-Saharan Africa, AGYW bear a disproportionate burden of HIV risk compared to their male counterparts. This coupled with high prevalence of sexual violence from IPs and non-partners, suggests that elevated rates of sexual violence may be compounding the burden for AGYW. Violence from non-partners significantly exacerbates poor health—it is associated with STI experience and increased anxiety and depression in both Kenya and Zambia. Sexual violence from IPs is also associated with health outcomes. In Kenya, sexual violence from IPs increased likelihood of STI experiences and higher levels of anxiety and depression. A previous systematic review on the health effects of non-partner sexual violence highlighted that it can lead to both short-term and long-term health consequences, especially mental health disorders, such as depression, anxiety, and alcohol abuse [2]. Previous research has also documented how gender-based violence can be a barrier to women’s engagement in HIV prevention and the treatment cascade. Our findings suggest that programmatic efforts should take into account violence from both intimate partners and non-partners and its influence on HIV prevention and care seeking behaviors, particularly for AGYW.

We also find that sexual violence from non-partners increased HIV risk perception, but the same was not true for sexual violence from intimate partners. Some qualitative work done as part of this study and other work in the region speaks to the pervasive nature of violence in youth relationships [4]. It is possible that young women may not perceive themselves at higher risk of HIV within their IP relationships, even when they experience sexual coercion, as they do if they experience sexual coercion from other men. How HIV risk perception may differ based on the perpetrators of sexual violence—whether intimate or non-intimate partner—warrants further attention.

Our findings suggest the importance of increased and more effective approaches to stem experiences of sexual violence for AGYW. Given the low uptake of post-violence care, increased routine screening for sexual violence from intimate and non-partners for AGYW could substantially aid in more effectively linking AGYW to post-violence counseling, support, and care. For instance, screening for violence at health facilities have been shown to be effective in facilities where quality services are provided [22–23] and could potentially be adopted for AGYW sexual violence programming.

Moreover, engaging with and addressing the underlying factors that perpetuate and exacerbate gender-based violence is a critical step for HIV prevention efforts [24]. Underlying gender norms (e.g., around masculinity and femininity), gender inequity (e.g., lack of legal protections or rights for women), and interpersonal power dynamics (e.g., sexual relationship power in age-disparate partnerships) need to be addressed for effective violence reduction and HIV prevention. Gender-transformative interventions, such as the Stepping Stones program engaging men and boys and women and girls to build gender-equitable partnerships for HIV prevention [25], should be expanded. At the same time, interventions that go beyond the changing perspectives and attitudes at the individual or interpersonal level to addressing community and structural level factors that perpetuate violence should be considered [16, 26–27]. Adaptation, implementation, and evaluation of successful targeted (e.g., school-based violence prevention for boys and girls) or comprehensive approaches (e.g., reaching community leaders, family, role models, male partners of AGYW, parents and guardians)—such as those being implemented by the DREAMS partnership—in underserved high burden HIV settings [28–30] could help break the cycle of violence against young women.

Our study is not without limitations. We rely on cross sectional data and are unable to investigate causal relationships between violence and health outcomes. We also rely on self-reported data—the experiences of violence and health outcomes—and thus, the data may be susceptible to a number of biases, such as social desirability and recall biases. Participants might over-report or under-report the experience of sexual violence, STI symptoms, anxiety and depression, and perception of HIV risk. There may also be some influence of stigma in under-reporting around HIV status, violence experiences, and types of partnerships. Finally, we did not examine sexual orientation or gender identity and how this may shape experiences of violence and HIV-related risk. Our study also points to the need for further examination of violence.

We also found some country-level differences in the association between IPV and health outcomes. While previous work in Zambia has found that experience of IP sexual violence was significantly associated with poor health seeking behavior and reduced adherence to prevention of mother to child transmission protocols [31], we are unable to ascertain why in our study young women’s experiences of sexual violence from IPs may not have the same association with health outcomes as in Kenya or in previous research. It is possible that AGYW in Zambia do not perceive sexual coercion from intimate partners as violence and are thus less likely to feel at risk of HIV acquisition or have greater anxiety or depression. This hypothesis, however, bears deeper investigation into the nature and perceptions of sexual coercion within intimate partner relationships in different country contexts. Also worth investigating are the specific characteristics of partners that perpetuate violence [32]. In this analysis, we did not collect, and are therefore unable to examine, the specific characteristics of intimate or non-partners from whom AGYW experienced violence. Information on the characteristics of partners who perpetrate violence and examination on context-specific understanding of sexual violence and coercion within intimate and non-partner relationships could have important programmatic implications.

Our analysis is focused on the relationship between sexual violence and sexual and mental health outcomes within the context of burgeoning HIV epidemics among AGYW. Broader investigations into the relationship between sexual violence and other short- and long-term impacts on AGYW’s lives may be warranted. For instance, subsequent analyses may also need to examine the linkages between experiences of sexual violence and pregnancy—a key experience for young women in these community contexts—or AGYW’s educational and employment outcomes, or the inter-generational impacts of sexual violence experiences during adolescence and young adulthood. Finally, while we expand upon sexual violence experiences for AGYW, additional work is needed to tease out sexual, physical, and emotional/psychological violence experiences among AGYW. Each of these may have a differential impact on health outcomes and are rarely measured and reported for AGYW in settings such as Kenya and Zambia.

In conclusion, sexual violence constitutes a significant public health problem in Kenya and Zambia. AGYW are at a higher risk of experiencing IP *and* non-partner sexual violence than previously reported. Experience of sexual violence is connected to poor sexual and mental health outcomes of AGYW. Strengthened sexual violence prevention programs, as well as expanded sexual violence screening and provision of post-violence care, are critically needed to reduce the high rates of sexual violence and related poor sexual and mental health outcomes of adolescent girls and young women.
